# Biliary Anastomosis Using T-tube Versus No T-tube for Liver Transplantation in Adults: A Review of Literature

**DOI:** 10.7759/cureus.24253

**Published:** 2022-04-18

**Authors:** Mallorie Vest, Camelia Ciobanu, Akwe Nyabera, John Williams, Matthew Marck, Ian Landry, Vikram Sumbly, Saba Iqbal, Deesha Shah, Mahmoud Nassar, Nso Nso, Vincent Rizzo

**Affiliations:** 1 Internal Medicine, Icahn School of Medicine at Mount Sinai, Queens Hospital Center, Jamaica, USA; 2 Medicine, St. Barnabas Hospital, New York City, USA; 3 Internal Medicine, New York City Health and Hospitals/Queens, New York City, USA; 4 Medicine, Icahn School of Medicine at Mount Sinai, Queens Hospital Center, Jamaica, USA

**Keywords:** biliary tract reconstruction, biliary anastomosis, biliary complications, biliary strictures, orthotopic liver transplantation

## Abstract

The T-tube-directed biliary anastomosis in orthotopic liver transplantation (OLT) aims to minimize preventable biliary complications, including bile leaks and strictures. Biliary complications in patients with OLT increase the risk of morbidity and mortality. This review paper evaluated the current evidence on the routine use of T-tube reconstruction in OLT cases.

A review of prospective, retrospective, observational, cohort studies as well as systematic reviews, meta-analyses, review papers, and opinion papers has been conducted to evaluate the therapeutic potential of T tube-based biliary anastomosis in cases of OLT.

Our finding showed a bile leak incidence of 16.6% and 6.6% in T-tube and non-T-tube groups, respectively. The results indicated a lower incidence of anastomotic fistulae in the non-T-tube group (0.6%) compared to the T-tube group (4%). The findings negated statistically significant differences in the three-year actuarial survival rates based on biliary anastomosis with and without T-tube intervention (62.5% vs. 69.8%). The studies revealed a 6-11% and 2-11% incidence of cholangitis in OLT patients with T-tube-based reconstruction and those without a T-tube, respectively, and 26% and 20% incidence of total biliary complications in OLT patients with and without T-tube, respectively. In addition, the findings ruled out the influence of a T-tube on the incidence of perioperative complications, endoscopies, and reoperations in OLT cases.

The current evidence correlates the increased incidence of bile leaks, cholangitis, and overall biliary complications with the use of a T-tube during OLT. In addition, T-tube-guided reconstruction has no impact on perioperative complications, overall survival, endoscopies, and reoperations in OLT cases.

## Introduction and background

The surgical treatment for end-stage liver diseases, including chronic liver disease and irreversible acute liver failure in adults warrants orthotropic liver transplantation (OLT) [1]. However, the surgical management of OLT requires postoperative monitoring to ascertain graft survival and minimize the risk of vascular complications. The medical management during the immediate postoperative period in OLT cases is challenged by several complications including arterial stenosis, resistive index/hepatic arterial velocity elevation, pleural effusion, perihepatic hematomas, pneumobilia, and hepatic edema [2]. Additionally, in hepatobiliary surgery, a T-tube is often used for achieving biliary anastomosis [3]. The primary benefit of biliary anastomosis in OLT is that it minimizes the incidence of preventable complications, including bile duct strictures and fistulas. However, the inconclusive evidence questions the potential of T-tube-based biliary reconstruction in minimizing bile leakage and biliary stricture [3]. The incidence of morbidity and mortality after OLT is predominantly attributed to biliary complications, including biliary strictures and leaks. The stenting of the biliary tract via T-tube during biliary tract reconstruction also aims to assess the color/flow of bile and reduce the risk of anastomotic strictures [4]. Moreover, T-tube insertion during OLT is not devoid of clinical complications. Recent evidence indicates a high incidence of T-tube-related cold ischemia time, cholangitis, and bile leaks in patients who undergo OLT. The reduction in T-tube-mediated biliary strictures at the cost of overall biliary complications challenges its routine utilization in biliary tract surgeries [5]. This review paper examined the current evidence on the routine utilization of T-tube in adult patients requiring OLT.

## Review

Methods

We utilized PubMed, Web of Science, Scopus, Embase, MEDLINE, Cochrane Library, and JSTOR to review studies that investigated the advantages, disadvantages, therapeutic benefits, and clinical complications after biliary reconstruction (with and without T-tube) in patients who underwent OLT. We focused on articles that discussed biliary strictures, biliary leaks, cholangitis, overall biliary complications (i.e., a composite of cholangitis, non-anastomotic/anastomotic strictures, fistula/bile leaks, Roux limb stasis/bleeding, biliary tract infection, and technical biliary complications), overall survival, re-surgeries, endoscopies, and perioperative complications in adult patients ≥ 18 years of age. We excluded studies that focused on primary sclerosing cholangitis, those with recipient-donor duct size mismatch, and where the recipient age was ≤ 18 years.

Biliary complications with liver transplantation

The potential intricacies reported after OLT include graft rejection, vascular complications, and biliary complications (regarded as the Achilles’ heel of liver transplantation) [6]. The morbidity and mortality incidence after OLT is predominantly due to biliary complications. The predominant causes of biliary complications in OLT cases include primary ductal disease recurrence, cytomegalovirus infection, chronic rejection, ABO blood type incompatibility, cold ischemia time, suture material, graft ischemia, and surgical anastomosis techniques, including T-tube utilization. In addition, 5-30% of patients with whole organ OLT experience biliary complications despite marked improvements in implantation techniques, immunosuppression procedures, and organ selection/preservation/retrieval [6]. The postoperative biliary complications predominantly occur after biliary reconstruction in OLT cases. Roux-en-Y hepaticojejunostomy and choledochocholedochostomy techniques are used for biliary reconstruction in OLT cases. However, choledochocholedochostomy is preferred due to its short operative time and presumed capacity to reduce the risk of biliary countercurrent infection. Choledochocholedochostomy also rules out the need for intestinal reconstruction and retains the biliary physiology by preserving the sphincter of Oddi.

T-tube indication in OLT

**Figure 1:**
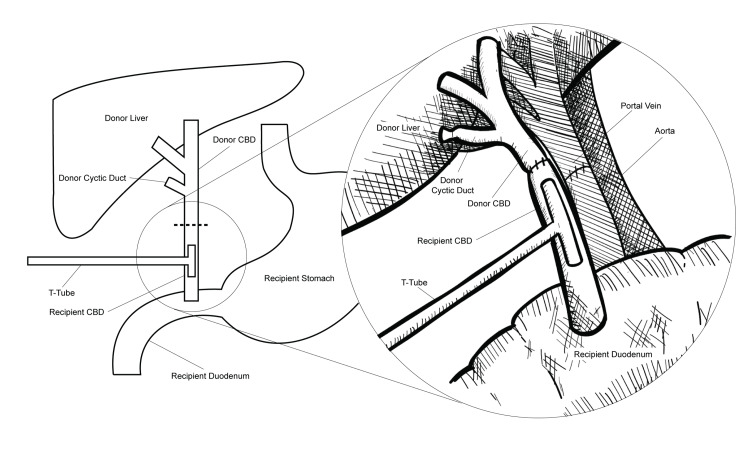
T-tube guided biliary reconstruction with choledochocholedochostomy. The image is created by the author (Matthew Marck) of this study. CBD: common bile duct

The liver transplant centers selectively utilize a T-tube to perform the choledochocholedochostomy reconstruction (Figure 1) [7]. The T-tube is used in the biliary system to reduce the risk/incidence of biliary complications by mechanically strengthening the anastomosis. However, the standard use of a T-tube in liver transplant cases is governed by several factors, including recipient conditions, graft quality, and technical complexity. The contemporary literature advocates the potential of a T-tube to minimize the severity of biliary complications and incidence of anastomotic strictures. Few studies substantiate the use of T-tube in liver transplantation in cases of duct caliber discrepancy and risky anastomoses. The use of a T-tube is also governed by the recipient’s risk of cold ischemia time prolongation and the model for end-stage liver disease (MELD) score [8]. Other factors include a high-risk donor (defined by pre-donation liver injury and advanced age), high-risk recipient, caliber of the bile ducts, and anastomosis complexity. The recipient surgical technique using a T-tube is used in patients who undergo total hepatectomy with caval preservation. The establishment of portal reperfusion depends on the flushing of the graft with Ringer’s lactate (50 cc) at 37°C [9]. The cholecystectomy follows the assessment of arterial/portal flow and arterial anastomosis, and the subsequent use of an Fr5 Argyle catheter (Minneapolis, MN: Covidien) on the cystic duct via the common bile duct of the graft. In addition, ductoplasty is undertaken to align the common bile duct and cystic duct in cases where they fail to communicate. The preparation of common bile is followed by its catheterization for patency/permeability assessment. The end-to-end anastomosis is undertaken to achieve T-tube-directed choledochocholedochal anastomosis. A running suture of 6-0 polydioxanone (PDS) is utilized to create a posterior anastomosis for placing the T-tube into the biliary system. The single stitches using 6-0 PDS are subsequently secured to construct the anterior face of the anastomosis. Cholangiography with the T-tube is finally performed to rule out clinical complications.

Advantages and disadvantages of T-tube in OLT

The use of T-tube during OLT helps preserve the physiology of the transplanted liver by assessing the quantity of bile [10]. It also facilitates the radiographic investigation of the biliary tree and safeguards biliary anastomosis by minimizing its intraductal pressure. The aim of T-tube utilization also correlates with the need to prevent non-anastomotic and anastomotic biliary strictures in OLT cases. In addition, it reduces the need for invasive diagnostic interventions in OLT patients with a high predisposition to fistula and bile leaks. A recently developed technique facilitates a tunneled biliary T-tube via the retroperitoneal path to minimize the risk of delayed healing and ascites exposure during OLT. This technique also helps minimize bile leakage, biliary peritonitis, and abdominal cavity spillage incidence after T-tube elimination. The contemporary literature also elaborates on the potential disadvantages of using a T-tube in OLT cases. The T-tube removal often triggers biliary leakage that adds to the incidence of cholangitis and biliary peritonitis, thereby predisposing the patients to severe morbidity [11]. In addition, T-tube placement adds to the risk and incidence of suture-based stenosis/insufficiencies. T-tube placement in OLT cases also predisposes the patients to infection-related morbidities due to the incidence of biliary leaks (27%) and intraabdominal sepsis (40%) [12]. However, high variability in the T-tube inherent complications in OLT patients lead to inconclusive evidence regarding their incidence after anastomotic stenosis. The clinical studies indicate the use of a T-tube in OLT cases with inconsistencies in the caliber of the bile duct. The OLT patients with a bile duct diameter of < 7 mm also require a T-tube for biliary reconstruction to reduce their risk of stenosis [13]. The rare complications associated with a T-tube in OLT cases include hemothorax and pancreatitis [14]. In addition, the currently reported T-tube-related biliary complications do not add to the incidence of subsequent surgeries and endoscopic interventions [5]. The absence/presence of Charcot’s triad and infection symptomatology rule out or confirm cholangitis in OLT patients after the use of a T-tube. However, magnetic resonance imaging, computed tomography, T-tube cholangiogram, endoscopic retrograde cholangiopancreatography, and percutaneous transhepatic cholangiography investigate biliary strictures/biliary leaks after OLT [1].

Complications with T-tube versus no T-tube in OLT 

Bile Leaks and Strictures

The prospective cohort study by López-Andújar et al. revealed the presence of anastomotic stenosis in 2% of OLT patients with T-tube compared to 12% in those without T-tube [7]. In addition, the resolution of T-tube-related biliary stenosis was achieved by percutaneous transhepatic cholangiography/endoscopic retrograde cholangiopancreatography-guided biliary prostheses placement and dilatation. However, the stenosis rates in the T-tube group were higher than in the non-T-tube group at one, three, and five years (2%, 16%, and 14% vs. 3%, 9%, and 20%, respectively). The findings revealed a lower incidence of anastomotic fistulae in the non-T-tube group (0.6%) compared to the T-tube group (4%). The results also confirmed a 3.3% incidence of anastomotic bile leak in the T-tube group compared to 0.6% in the non-T-tube group. The randomized study by Scatton et al. indicated a 1.11% incidence of T-tube-related anastomotic strictures in the T-tube group compared to an incidence of 4.44% in the non-T-tube group [14]. The retrospective observational study by Ong et al. revealed a 12.6% incidence of anastomotic strictures after the use of a T-tube compared to 5.3% in patients without a T-tube [15]. In addition, an anastomotic leak was recorded in 2.3% of patients with a T-tube compared to 2.6% in patients without a T-tube. The retrospective study by Cantero et al. indicated anastomotic stenosis in 22.2% of patients with a T-tube compared to 80% in patients without a T-tube [16]. The bile leak incidence was recorded as 16.6% and 6.6% in T-tube and non-T-tube groups, respectively. The clinical studies reveal a 2-25% incidence of bile leak within one to 180 days after OLT. In addition, an observed 6-12% incidence of anastomotic strictures based on implant type, duct-to-duct anastomosis, reperfusion injury, and ischemia impacts the postoperative management in OLT cases [17]. The anastomotic bile leaks potentially increase the risk of biliary strictures. The cystic duct remnants, Luschka’s duct, percutaneous transhepatic cholangiography tube tract, and anastomosis often trigger bile leaks after OLT. In addition, bile duct strictures attribute to 40% of postoperative biliary complications in patients with OLT [18]. 

Overall Survival

The contemporary literature reveals a higher incidence of overall complications in OLT patients with T-tube-based biliary anastomosis (33%) compared to patients without T-tube-directed reconstruction (15.5%) [19]. The findings correlate the lower survival rate (72.8%) with T-tube-related complications in OLT cases. In contrast, a higher survival rate is recorded in patients who undergo OLT without T-tube-guided biliary anastomosis. However, these results cannot be generalized in all OLT cases since clinical studies also negate statistically significant differences in three-year actuarial survival rates based on biliary anastomosis with and without T-tube intervention (62.5% vs. 69.8%) [14]. The inconsistencies in survival rates attribute to median delays in biliary complications after OLT (i.e., 111 days in non-T tube cases vs 38 days in T-tube-directed reconstructions). 

Cholangitis

Cholangitis in OLT cases develops under the impact of serious biliary obstruction caused by the injured bile duct walls and bile thickening that potentiates the formation of biliary sludge [20]. The clinical complications including bile stagnancy/stasis, ischemic-type biliary lesions, biliary strictures, and liver graft ischemia lead to the development of biliary casts. Cholangitis also increases the risk of bile leaks after T-tube removal or dislodgement following OLT. In addition, 5-20% of OLT patients develop primary sclerosing cholangitis in the postoperative tenure [20]. The male patients and patients with an intact colon before OLT remain predisposed to cholangitis recurrence. The differential diagnoses to rule out fibrous cholangitis include biliary cirrhosis, biliary fibrosis, ductopenia, fibro-obliterative lesions, ischemic-type biliary lesions, and hepatic artery stenosis. However, the recurrence of cholangitis does not deteriorate the graft and patient survival after OLT. Clinical studies reveal 6-11% incidence of cholangitis in OLT patients with T-tube-based reconstruction compared to 2-11% in patients without T-tube [9,14,21-23].

Overall/Total Biliary Complications

The biliary complications following OLT occur due to several causes, including imperfect duct-to-duct anastomosis, the use of a T-tube, infections, immunologic injury, ischemia-reperfusion injury, and hepatic artery stenosis [20]. In addition, 10-30% of biliary complications add to the 10% mortality rate in patients with OLT [20]. The bile duct obstruction after OLT occurs due to several intrinsic complications including, biliary stones/cast/sludge, T-tube remnants, nematodes, and thrombi in hemobilia. The extrinsic complications include biloma, abscess, hematoma, false aneurysm, cystic duct mucocele, de novo/recurrent cancer, and posttransplant lymphoproliferative disease. The hepatic artery thrombosis predominantly triggers ischemic biliary complications, including biliary abscess, biloma, leaks, and stricture, after OLT. The technical biliary complications include, cystic duct mucocele, kinking, missed segmental duct leak, cut surface leak, T-tube-based reconstruction, and anastomotic leak/stricture. The ischemia type biliary lesions develop due to immunologic, idiopathic, and ischemia-reperfusion injury-related factors. Other biliary complications include cholangitis, biliary tract infection, and Roux limb stasis/bleeding. The clinical studies indicate a 26% incidence of total biliary complications in OLT patients with T-tube-directed biliary anastomosis compared to 20% in non-T-tube cases [3,7,9,14,15,21,22,24-30].

Reoperations or Repeated Surgeries/Endoscopies 

The re-operative interventions or endoscopies in OLT cases are predominantly attributed to postoperative hemorrhage or intraoperative blood loss [31]. Other potential causes of re-operations/endoscopies in OLT cases include anastomotic/non-anastomotic leakage, biliary abscess, biloma, anastomotic/extra-anastomotic stricture, intrahepatic stricture, papillary dyskinesia, bile stones, bilioenteric anastomosis, mucocele, and hemobilia [32]. In addition, 30-50% of re-transplantations are attributed to graft loss due to the non-anastomotic strictures and failure of endoscopic interventions [33]. The clinical studies indicate 0.6-0.7% incidences of reoperations in OLT cases irrespective of T-tube-guided biliary anastomosis status [5]. 

Perioperative Complications

The perioperative complications in OLT cases impact the recovery process and increase the risk of morbidity and mortality. The potential indicators of rehospitalizations based on perioperative complications include operative time, the MELD score, warm ischemia time, and hepatic artery thrombosis [5]. The literature findings to date do not reveal significant differences in perioperative complications between T-tube recipients and patients without T-tube-guided reconstruction. However, the use of T-tube significantly increases the risk of long-term complications (including Kaposi’s sarcoma, renal failure, acute/chronic rejection, osteoporosis/osteopenia, recurrent liver disease, infections, and metabolic complications) that deteriorate the prognostic outcomes and reduce the survival time [34].

The Current Recommendation for T-tube Utilization in OLT

Proper identification of the causes of anastomotic biliary complications after OLT is conducive to improving the therapeutic outcomes and reducing the risk of resultant morbidity and mortality. The literature findings to date do not indicate any statistically significant differences between adversities caused by different biliary reconstruction methods including, T-tube-related choledochocholedochostomy, cholecystojejunostomy, gallbladder conduit, and cholecystoduodenostomy [35]. The current evidence on the effect of these interventions on morbidity and mortality restricts their routine administration during liver transplantation. However, T-tube-directed reconstruction in OLT cases aims to facilitate biliary tree monitoring through radiographic imaging. The surgeons continue to presume the role of T-tube in safeguarding biliary anastomosis by minimizing the intraductal pressure. However, the latest evidence is inconclusive concerning the role of T-tube-directed biliary anastomosis in minimizing the risk of biliary strictures [1]. Several clinical studies confirm a high incidence of overall biliary complications (including bile leaks, strictures, and cholangitis) in OLT patients with T-tube-guided reconstruction. In addition, the prolonged placement of T-tube increases the risk of non-anastomotic biliary strictures by potentiating fibrosis and inflammation in the biliary system. The deterioration of the local host defense mechanism due to T-tube increases the risk of infection and bile leaks that eventually trigger anastomotic biliary strictures after OLT. The T-tube utilization in OLT provides no advantage in terms of reducing the risk of perioperative complications, endoscopies, and re-operations [5]. It also does not provide a survival benefit to patients with OLT. Therefore, these findings do not advocate the routine utilization of T-tube for biliary anastomosis in OLT. 

## Conclusions

T-tube-directed biliary reconstruction in OLT remains debatable due to the inconclusive evidence on its postoperative therapeutic benefits. The limited evidence favors the reduction in bile strictures after T-tube utilization for duct-to-duct anastomosis. However, the current evidence indicates a higher incidence of overall biliary complications, biliary leaks, and cholangitis in OLT patients who receive the T-tube placement for biliary anastomosis. In addition, the use of T-tube does not provide a survival benefit and has no significant role in minimizing the incidence of perioperative complications. The T-tube-guided anastomosis also does not impact the risk of endoscopies and reoperations in OLT cases. Therefore, these findings negate the routine use of T-tube for biliary anastomosis in liver transplantation.
